# Mapping a high‐level overview of information flows in the Dutch declaration chain for medical specialist health care

**DOI:** 10.1111/hir.12334

**Published:** 2020-11-17

**Authors:** Philippe van der Voorn, Nico Brand

**Affiliations:** ^1^ Utrecht University Utrecht Netherlands

**Keywords:** health records, information management, public health sector

## Abstract

This study is based on Philippe van der Voorn’s master’s dissertation at the Utrecht University, Department of Science, Information and Computing Sciences. The problem identified was a lack of an integrated information chain and clear governance structure for information flow in the Netherlands’ health care sector. The method of Design Science was followed to construct an overview model of the chain, and towards a business process model that is intuitive for both technical and business users. An initial declaration chain was identified in the literature and presented, to be confirmed and elaborated on via eight interviews at seven different organisations in the medical specialist health care sector. Based on these interviews, the draft overview was adjusted and a Business Process Model and Notation model created that indicates the shared understanding of the data elements and activities between the organisations. The contribution of the overview of the declaration chain, in particular, can help medical specialist staff obtain an understanding of the administrative side of their work, and with a clear information infrastructure lead to better working processes and information quality. F.J.

## Introduction

Health care in the Netherlands is not an integrated chain but a collection of individual entities including hospitals, health insurers and regulators, and yet a clear governance structure and architecture is missing for information flow between these entities, Kennisgroep Keteninformatiemanagement (2016). As a result, information flows are complicated, is not directed in its entirety and it is unclear where exactly the responsibilities and powers lie and who is addressed to what (Kennisgroep Keteninformatiemanagement, 2016; Plotkin, 2014). To illustrate, who is responsible for the registration of the age of a patient within the health care chain; is it the general practitioner, the hospital, the insurance company or the government?

These problems are primarily occurring in the so‐called interorganisational networks or chain computerisation perspective. There are many definitions of interorganisational networks in the scientific literature. Essentially, at the foundation of all definitions lies the concept of networks consisting of the structure of relationships between actors (organisations and individuals), the essence of the links between actors and the meaning of those relationships (Popp et al., 2014).

The consequences of a lack of an integrated information chain is that it is hard to rely on the quality of the information provision in the health care sector, but information quality is of great importance. A good information infrastructure makes sharing information between entities more efficient and effective, which leads to a better working process (Kennisgroep Keteninformatiemanagement, 2016; Welters, 2016). The problem is that there is no such complete model of the declaration chain and information flows of the medical specialist health care in the Netherlands. Our aim is to visualise and improve on an initial information flow model via validation interviews. With these models, administrative workers in hospitals and doctors can get a better understanding why it is important to correctly and timely register all the health activities that are performed. During our interviews, it was clear that currently, this understanding was not yet there.

This paper is structured as follows: first, the research methodology will be explained followed by background research that will highlight the current literature about the Dutch declaration chain. In the next section, the results are elaborated in which a better overview of the chain is presented, and the model is validated by experts. Finally, the findings are discussed and concluded, and future directions are given.

## Methods

**Figure 1:**
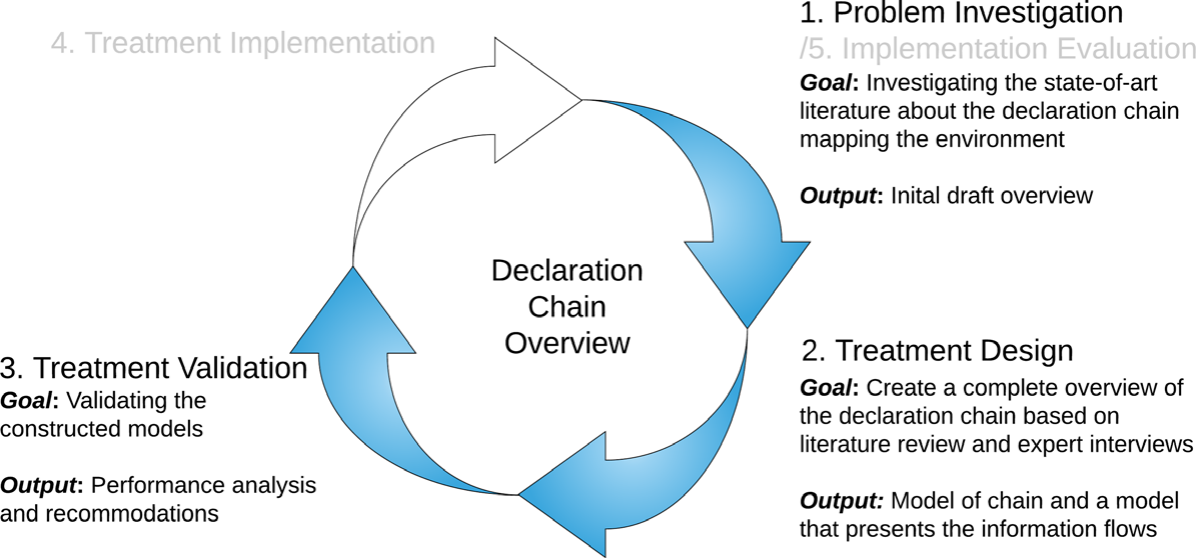
Design Science Methodology for this research

Within this research, a new object (artefact) is constructed to improve the problems as mentioned earlier (context). The artefact is iteratively investigated and designed using existing and newly gained knowledge. This study will, therefore, be characterised as Design Science (Wohlin & Aurum, 2015). We follow the method of Design Science from Wieringa (2014). This method is created to solve a problem by creating an artefact. In this research, we will use the Design Science Cycle (Figure 1) for creating an overview of the declaration chain of medical specialist health care in the Netherlands in a chain computerisation setting.

The Design Science Cycle contains five phases: problem investigation, treatment design, treatment validation, treatment implementation and implementation evaluation. Due to limited time, we will only execute the first three phases of the Design Science Cycle.

To get an understanding of the Dutch declaration chain, a background study was performed. This is the problem investigation phase. In this background study, the initial draft declaration chain is presented which is to be further elaborated on via the interviewing of involved parties. Thus, the initial model will be the input for the expert interviews, which will be conducted at the participating organisations in the chain. Based on the interviews, the draft overview is adjusted to their comments and remarks, which is the treatment design phase. This final model is then validated by two more expert validation interviews to test whether the model is correct and investigate the added value, which is the treatment validation phase.

## Background

**Figure 2:**
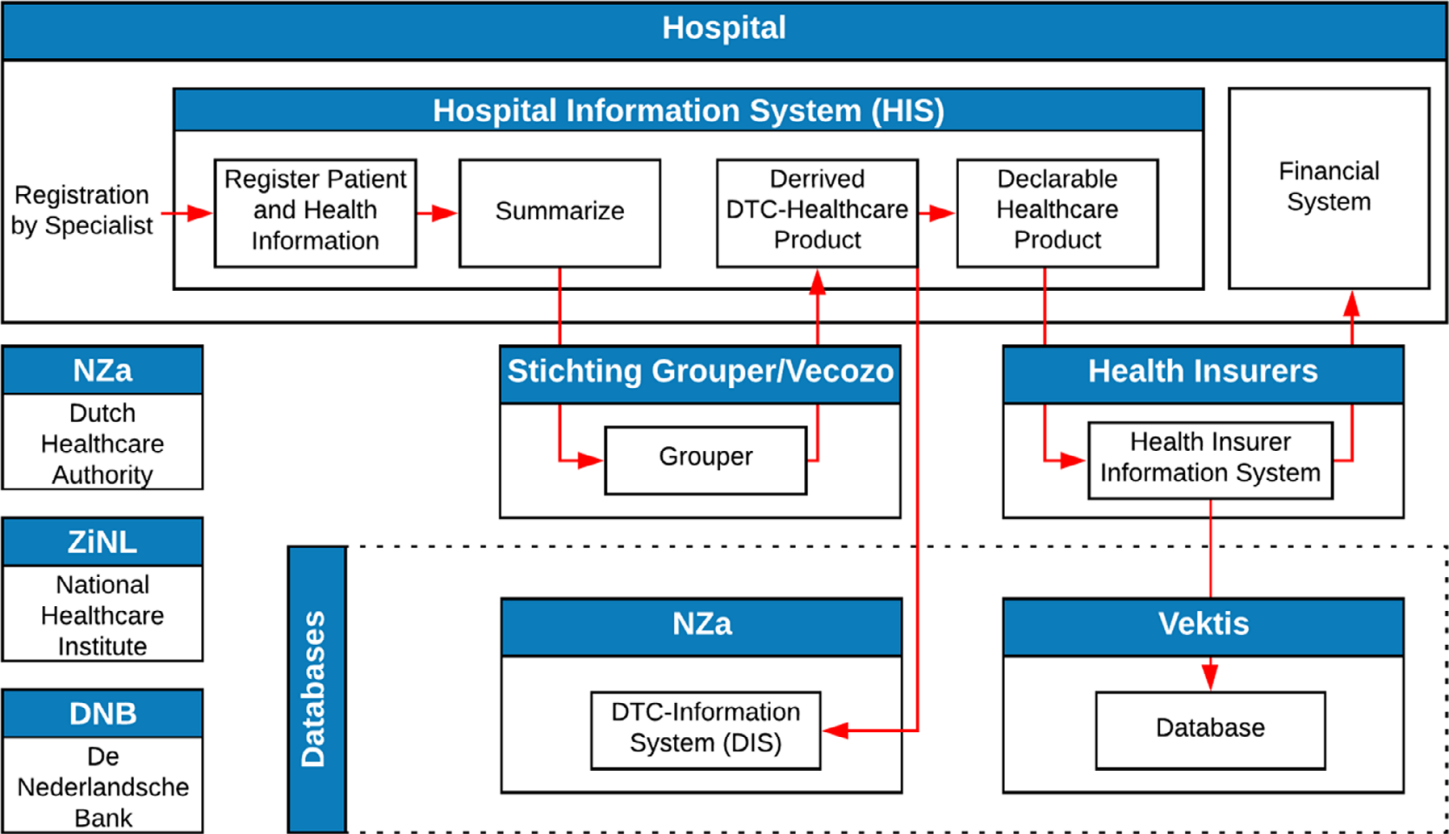
Schematic overview of the declaration chain derived from Kennisgroep Keteninformatiemanagement (2016)

Kennisgroep Keteninformatiemanagement (2016) created a schematic overview of the declaration chain in the health care sector. The overview shows the main flows that exist within the sector with health care providers and health insurers, and the most essential entities around it (Figure 2). However, this overview is already four years old and not complete. For example, the patient is not included, and there are more flows between the organisations that are not visible. Furthermore, the Grouper is divided into two separate components with which hospitals are communicating, and there are loops back from the hospital to the insurer.

The hospital starts where the specialist registers the patient were DTC stands for ‘Diagnosis Treatment Combination’ a nine‐digit code that says something about all activities and operations that a patient goes through within the care during a set period (Zorgwijzer, 2019). All services that can be claimed for money are expressed in so‐called DTC health care products, and there are approximately 4400 DTC health care products in the Netherlands. Recording takes place in the Hospital Information System (HIS), the care systems and financial systems of a hospital, and this includes the diagnosis of a specialist, hospital treatments and follow‐up checks.

Vecozo and Stichting Grouper draw up rules and standards for communication between chain parties in health care concerning the administrative handling of transactions. Within the administrative care domain, Vecozo facilitates a digital environment in which chain parties can exchange data with each other quickly, easily and securely. The Grouper lead a set of transactions. Via the decision tree of the Grouper, the transactions are grouped into individual DTCs with the price associated with this set of transactions. These DTCs are sent back to the HIS to be entered as a declaration.

Health insurers receive invoices that are sent by the hospitals, after which they are paid to the insured person after many checks (formal check, appropriate use, fraud, etc.) ZiNL (Care Institute the Netherlands) oversees the quality, accessibility and affordability, these being the pillars of the Dutch health care system. Care Institute the Netherlands has an important position in this system: they ensure that these pillars form a strong foundation. From each declaration paid by a health insurer, some data are sent to vektis and recorded in their database. vektis delivers this information about declarations of health care back to the health sector. vektis analyses the use, costs and quality of care based on all care declarations and insured person data. This provides support for decision makers in health care when making choices to maintain the quality and affordability of health care. De Nederlandsche Bank, as an independent central bank and regulator, is responsible for, among other things, a stable financial system, a safe and efficient payment system and, regarding health insurers, for financial institutions to meet their obligations.

In the next section, expert interviews will review the above declaration chain from Kennisgroep Keteninformatiemanagement (2016) and evaluate how this overview can be transformed towards a complete model that includes the missing elements identified at the beginning of this section.

## Results

The interviews took place at seven different organisations and with ten people in various roles/functions (as shown in Table 1). The interviews aimed to capture the perspectives of how the declaration chain is functioning in the participating organisations in the chain. The interviewees were presented the initial overview of the declaration chain of Figure 2 and were asked whether it is correct and how it can be improved.

**Table 1:** Overview of the organisations and interviewees

Organisation	Function	Size
Health Insurer	Manager Internal Audit, IT Auditor and Operational Auditor	2500
Health Insurer	Manager Internal Audit	2500
Vecozo	Business Manager	200
Hospital X	Head of Health Control	1100
NZa	Advisor Information Management	400
ZiNL	Team Manager Data Management	400
Hospital Y	Staff Advisor Health Registration	12 000
vektis	Head of Compliance, Audit & Risk	120
Hospital Y	Coordinator Health care Purchasing Planning & Control	12 000
Hospital Y	Head of Invoicing and Debtor Management	12 000

The interview data were transcribed and processed in NVivo 12 a qualitative data analysis software package (Edhlund & McDougall, 2019). Each interview schedule was the same although small modifications were made per different organisation to gain more in‐depth information about their processes. For compatibility and easy referencing, NVivo allows coding the interviews on important topics.

The main findings from the interviews identified that Stichting Grouper is divided into two platforms, the grouper and declaration portal. Also, that the Ministry of Health, Welfare and Sport needed to be added to the chain (but due to time constraints and feasibility, not followed for an interview).

The organisations in the chain indicated that they are not aware of how exactly information is flowing between the organisations in the whole chain. It was clear they were only concerned with their part of work in the process, and not what other organisations are precisely doing. Furthermore, the patient was not visible in Figure 2 but as an outcome of the interviews is now added with the correct information flows.

**Figure 3:**
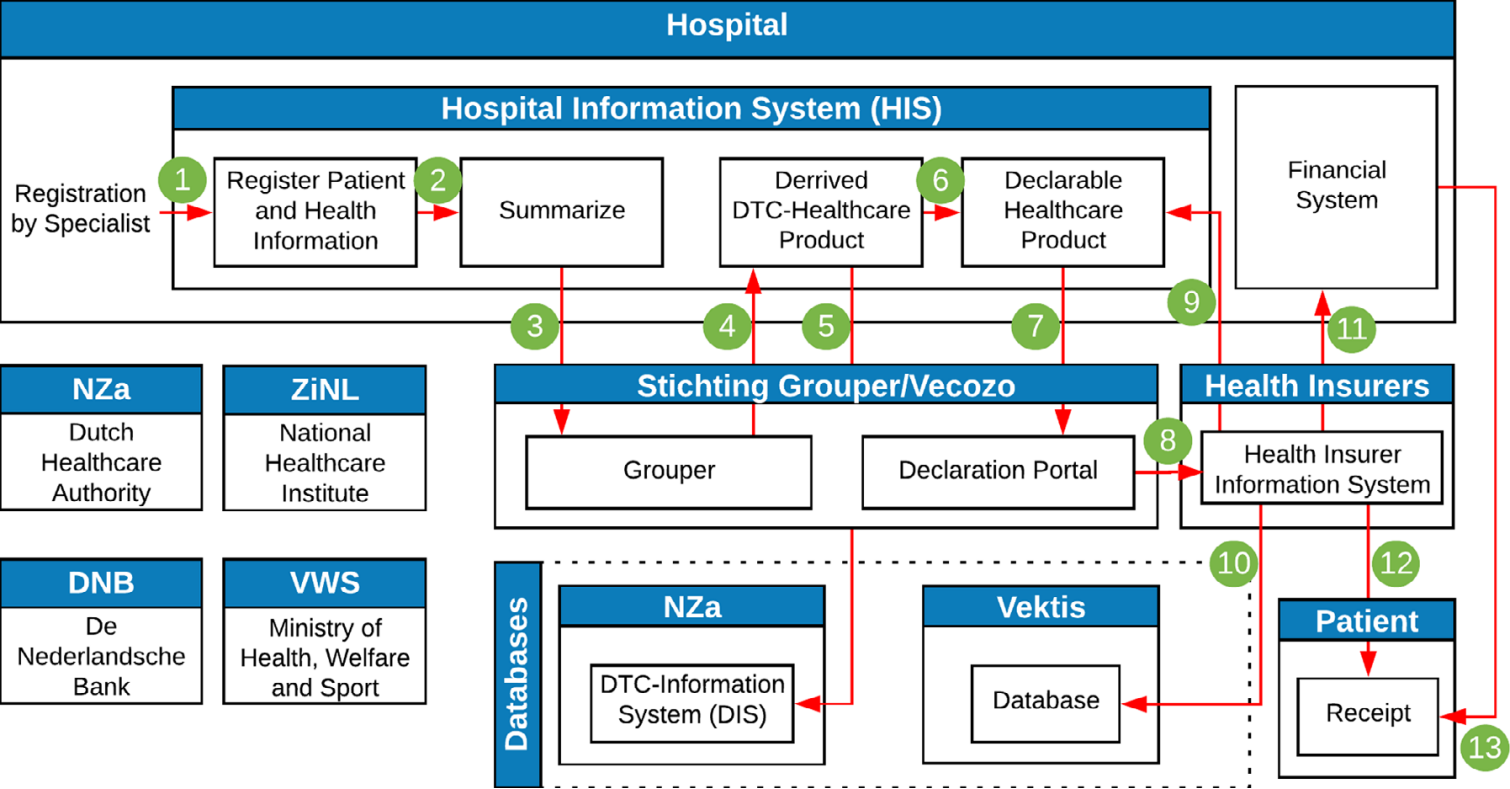
Schematic overview of the declaration chain improved via interviews

The new improved model is presented in Figure 3 and creates a foundation for a mutual understanding of the information flow with the different connections elaborated on with their corresponding number below the Figure. The interview findings were useful in developing this model since the identified flows cannot be found in the literature, while in practice, they exist. The practitioners gave their expert opinion to present the chain as completely as possible. They all had a mental picture in their heads how the chain is shaped but is not visualised in a document. With these interviews, an attempt is made to have this chain correctly visualised.

1. Medical specialist (or someone who has permission) registers information about the patient and performed health care activities;
2. All health activities are combined and grouped into a declaration dataset;
3. The declaration dataset is sent to the Grouper, for hospital X this happens every Wednesday afternoon;
4. The Grouper returns a health care product to the hospital;
5. Dutch Healthcare Authority receives around every two months a data dump from hospitals based on the output of the Grouper;
6. Each hospital has a price for each health care product that will be linked to the health care products;
7. Declaration from hospital is sent to the Declaration Portal of Vecozo;
8. Via the Declaration Portal, the declaration will go to the health insurer;
9. The declaration can get rejected by the insurer, and it goes back to the hospital for control. Adjustments can be made in the declaration so that the insurer will accept it;
10. Every health insurer sends monthly information about all their declarations to the vektis Database for analysis;
11. If the declaration is approved by the insurer, the hospital gets paid;
12. A patient will receive a receipt of the insurer that contains information about the own risk and costs;
13. Some medical activities will not go via the insurer, but the receipt goes directly to the patient. In most of the cases, this is for uninsured health care like plastic surgery for non‐medical reasons.

**Figure 4:**
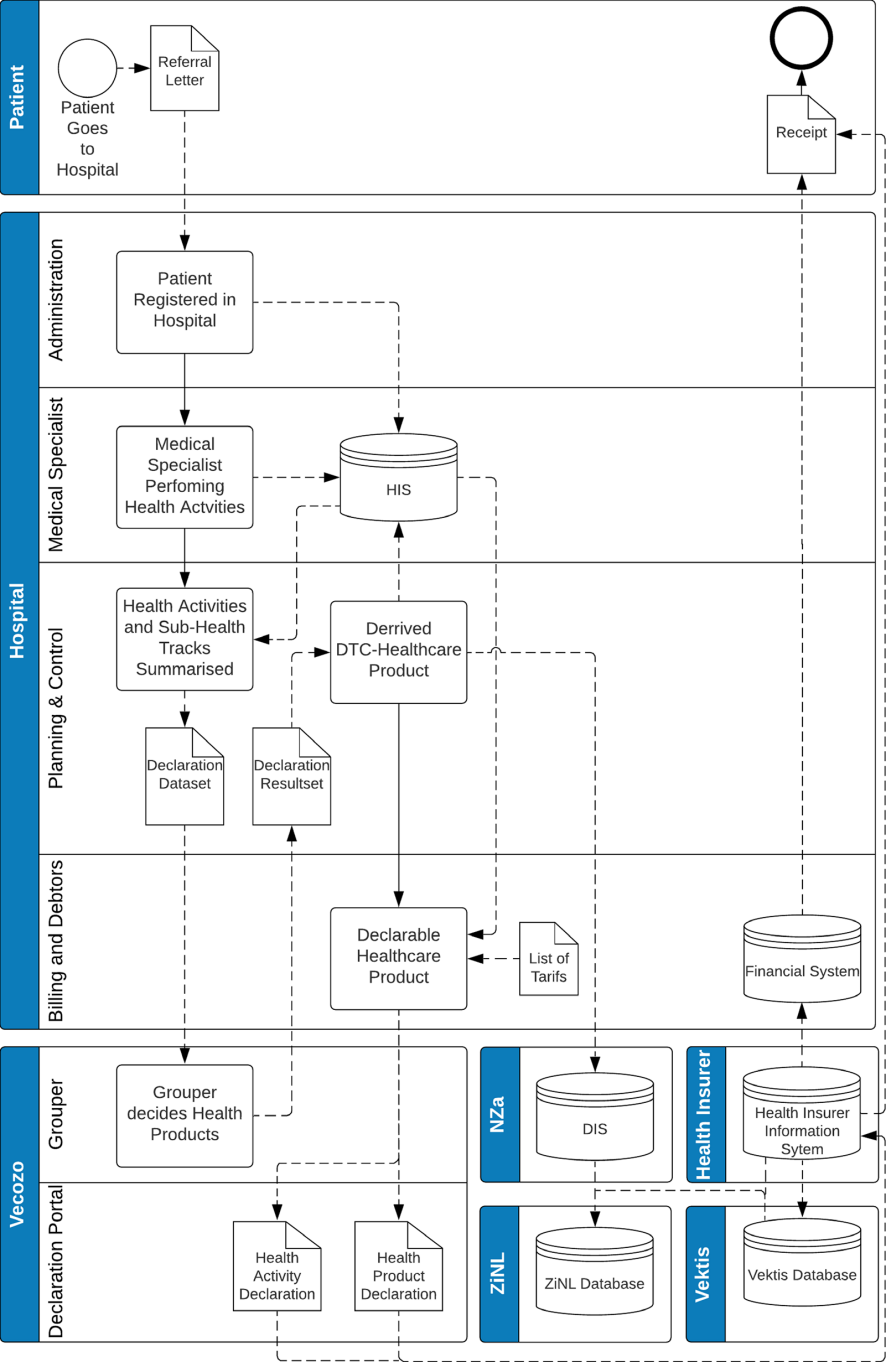
BPMN of the declaration chain with data elements and overview combined

Based on this information, the overview was transformed towards a Business Process Model and Notation (BPMN) model. The objective of BPMN is to support business process modelling for both technical users and business users, by providing a notation that is intuitive to business users, yet able to represent complex process semantics (Rosing et al., 2015). The BPMN presents how the different activities correlate with the data elements that flows between the organisations and is shown in Figure 4. The data elements in the declaration chain are derived based on the information by Nederlandse Zorgautoriteit (2020a, 2020b) and Federatie Medisch Specialisten (2019).

### Model validation

For the last part of the Design Science Cycle, the model needs to be validated. Two expert validation interviews were performed. The interviewees were the staff advisor health registration from hospital Y and a senior manager health care of a large accounting organisation.

In the BPMN, the information flows between and within the organisations are shown. This overview creates valuable information for the organisations in this declaration chain, according to the interviewees. The staff advisor health registration said, ‘I find the overviews very clear and visual, so it is definitely an added value’.

Furthermore, the senior manager stated that ‘the model can help the medical specialists because they never really understand why registration is complicated; they have to make people better and not do administration work’. This model does not provide insight into the complexity that happens behind the registration scenes. The manager of the accounting organisation had experienced this in practice when he was a manager in a health institution. To show how the registration process works, he invited health care professionals to look at the administration process and the other way around, let administrative employees follow the health care professionals in their daily work to create a mutual understanding of both sides. Furthermore, the manager argued that this model would help make the process leaner and can gain insight how it can be more efficient.

## Implications for practice

This research results in multiple contributions. The practical contribution of this research is that it creates a clear overview of the declaration chain created for medical specialist health care with their information flows. There was not such complete overview available, but now it can be used as a global picture for participants within the declaration chain or people who are exploring this. During the validation interviews, one important key finding came up and that is, with the created model, the doctors can get an understanding of how the administrative side of their work is functioning. Doctors do not want to do administrative work, but it is important to register all the health activities correctly; otherwise, there is a chance that the hospital will receive less money for the diagnosis. What can result in losing money, can eventually result in financial problems.

A threat regarding generalisability is that the created models only work for health care in the Netherlands, and health care in other countries are organised differently (Busse et al., 2013). However, a strength of the research is that the selected population for the declaration chain is fully covered by all the interviewed organisations. We looked at the declaration chain of medical specialist health care, but this chain is very similar to the chain of mental health care. The validation interviews indicated that the created model easily could be transformed into a model for mental health care.

## Conclusions

Derived from literature and eight interviews with all the participants of the declaration chain, we have identified how the high‐level overview of the chain is visualised and what the information flows between the organisations are.

The interviewees proposed different recommendations for the model to improve the level of detail and correctness. These improvements were added to model the current situation of the declaration chain, providing a correct high‐level overview.

The focus was on medical specialist care, while, as we identified, there are more different disciplines. Each has its declaration procedures that are different from our investigated medical specialist care. For these disciplines, their chain collaboration and data elements can be analysed to conduct analyses over the whole Dutch health care sector eventually.

This research provides a lot of different directions for additional research. In light of the current COVID‐19 crisis, the health system is under high pressure. Intensive cares are full, and many transportations of intensive care patients to other hospitals are occurring daily. For future research, it would be interesting to see what the impact of the COVID‐19 crisis has had on the registration processes. Do doctors still register all the products, or are they only concerned with helping patients? This can have consequences on the money coming in for hospitals which result in more financial pressure. A possible research question in this area is: How did the chain collaboration change in the health care sector focusing on intensive care due to the impact of the COVID‐19 virus?
